# Functional Characteristics of Lactic Acid Bacteria In Vitro Isolated from Spontaneously Fermented Sour Porridge with Broomcorn Millet in Northwestern Shanxi Province of China

**DOI:** 10.3390/foods11152353

**Published:** 2022-08-06

**Authors:** Qi Wang, Jiaqin Liu, Jin Cai, Sanhong Fan

**Affiliations:** 1 School of Life Science, Shanxi University, No. 92 Wucheng Road, Taiyuan 030006, China; 2 Institute of Applied Chemistry, Shanxi University, No. 92 Wucheng Road, Taiyuan 030006, China

**Keywords:** lactic acid bacteria, probiotics properties, sour porridge, broomcorn millet

## Abstract

Eighteen strains of lactic acid bacteria were isolated from spontaneously fermented sour porridge with broomcorn millet in Northwestern Shanxi Province of China, and their probiotic characteristics were investigated in vitro. Survival rates under gastrointestinal conditions, cholesterol reduction, antibacterial capabilities, antioxidant activities, and safety assessments were examined. Results showed that five strains were selected as probiotics and identified as *Levilactobacillus* *brevis*. Strain L10 exhibited excellent probiotic characteristics, with an 86% survival rate under pH 2.0 for 2 h, 80% survival rate in 0.3% bile salt for 6 h, the highest survival rate (78%) in simulated gastrointestinal juice for 3 h, the highest hydrophobicity (42% to xylene and 39% to hexadecane), the highest aggregation (39% auto-aggregation and 10.4–18.13% co-aggregation), relative higher cholesterol reduction rate (80%), the highest antibacterial activities, the highest antioxidant activity, sensitive to most antibiotics tested, without hemolytic and hydrolyze gelatinase activity and could not produce biogenic amine. Therefore, strain L10 could be applied to functional foods.

## 1. Introduction

Probiotics are defined as “live microorganisms that, when administered in adequate amounts, confer a health benefit on the host” (FAO/WHO, 2002). Probiotics have a symbiotic relationship with the host [1]; they could produce some nutrients or antimicrobial substances [2], lower serum cholesterol levels [3], prevent diarrhea [4], anti-cancer [5], anti-mutate [6], enhance immune response [7,8], produce antibiotics [9,10], modulate the enteric central nervous system [11,12,13], and so on.

Probiotics are common in many kinds of fermented foods, such as cereal, yogurt, cheese, sour milk, dry-fermented sausages, and so forth [14,15,16,17,18]. Traditional fermented foods were potential sources of probiotics as they were often composed mainly of lactic acid bacteria (LAB), including *Lactobacillus*, *Pediococcus*, *Enterococcus*, *Weisella,* and *Leuconostoc* [4]. In fermented foods, LAB produces copious amounts of metabolites such as lactic acid, acetic acid, ethanol, carbon dioxide, fatty acids, and antimicrobial peptides, which are responsible for flavor, texture, and preservation [19,20,21]. Furthermore, LAB could release kinds of bioactive compounds to improve the nutritive values of fermented foods. These bioactive compounds have many health-modulating effects such as anti-infection, immunomodulatory, anti-allergenicity, anti-obesity, antioxidant, and anti-anxiety, enhancing the bioavailability of vitamins/minerals [22,23,24,25,26,27,28,29], etc.

Fermented cereal-based foods are acknowledged to have higher nutritional value, longer shelf life, and are easier to digest compared with non-fermented cereal-based foods [4]. Broomcorn millet (*Panicum miliaceum* L.) is a small-seeded grain that has a long cultivated history and is widely planted as a staple food in Northern China. Broomcorn millet was one variety of millet [30], and millet has a shorter growing season and could resist drought, diseases, and pests compared to major cereals. The nutritional potential of millets in terms of protein, carbohydrate, and energy values are comparable to the popular cereals such as rice, wheat, barley, or bajra [31], and their importance as an ingredient in multigrain and gluten-free cereal products has been highlighted [30]. Broomcorn millet is high in protein content (>14%), with superior quality and optimum balance of essential amino acids [32]. It is a suitable foodstuff for patients with a gluten-free diet because the specific prolamin fraction is under the permitted level [33]. Its necessary amino acids (leucine, isoleucine, and methionine) are higher than in wheat [34].

Sour porridge is a kind of fermented cereals foods, which was family-style spontaneously fermented with broomcorn millet. It was consumed as a daily food in western Inner Mongolia and northwestern Shanxi Province in China. Although a large quantity of LAB was isolated from fermented cereals, and many of them were demonstrated with diverse functions [35,36,37], the functions of LAB were distinguished among various fermented cereals foods due to different raw materials, climates, and operation modes. Therefore, it is necessary to detect the probiotic characteristics of LAB isolated from spontaneously fermented sour porridge with broomcorn millet. The results could not only achieve potential probiotics but also could supply theoretical support for the exploration of sour porridge as a functional food.

## 2. Materials and Methods

### 2.1. Sour Porridge

Broomcorn millet was purchased in the local market and mixed with water in a tin (1:3, *w*/*v* ratio). The mixture was naturally fermented in the local family of Northwestern Shanxi Province of China. After several hours, the mixture was fermented into sour porridge. The sour porridge was stirred thoroughly, and 10 g were collected in triplicate and stored at 4 °C for analysis as soon as possible.

### 2.2. Strains

#### 2.2.1. Isolation of LAB for Probiotic Characteristics Evaluation

One gram of sour porridge was put into a conical flask containing 9 mL of sterile sodium chloride (0.9%, *w*/*v*) covered with glass beads. Then the slurry was shocked for 30 min in a shaker at room temperature, followed by centrifugation at 2500× *g* for 15 min. The supernatant was collected, serially diluted, and 1 mL of the suspension from 10^6^ dilution was plated onto the sterilized Man Rogosa Sharpe (MRS) [38] agar medium (Sangon). The plates were cultured at 30 °C for 1–2 days. Individual colonies were selected and purified. The morphological and physicochemical properties of all pure isolates were analyzed using Gram staining test, endospore test, cellular morphology and colony characteristics, biochemical tests (i.e., catalase, oxidase, nitrate reductase, arginine, amygdalin, etc.), the potentiality to ferment various saccharides such as sucrose, mannitol, glucose, arabinose and so on. All isolates were maintained at −80 °C in MRS broth supplemented with 20% (*v*/*v*) glycerol (Sangon).

#### 2.2.2. Indicator Pathogens for Co-Aggregation Determination

Indicator pathogens were G^+^ pathogens (*Listeria monocytogens* ATCC7644 and *Staphylococcus aureus* ATCC 15923) and G^−^ pathogens (*Escherichia coli* O157:H7 1934 and *Salmonella typhimurium* 02-8423) [39]. They were received from Shanxi Provincial Center for Disease Control and Prevention and conserved on Brain Heart Infusion media at the School of Life Science of Shanxi University in China.

#### 2.2.3. Indicator Strains for Antibacterial Activity Determination

Indicator strains were *Bacillus cereus* MTCC 430, *Escherichia coli* ATCC 11229, *Enterococcus faecium* ATCC 35667, *Listeria monocytogens* ATCC7644, and *Staphylococcus aureus* ATCC 11632 [17]. They were achieved by the Shanxi Provincial Center for Disease Control and Prevention and maintained on Brain Heart Infusion media at the School of Life Science of Shanxi University in China.

### 2.3. Survival of LAB in Simulated Gastrointestinal Conditions

#### 2.3.1. Tolerance to Acid

Acid tolerances were determined as the method described by Liong and Shah [40]. Each strain overnight grown culture was incubated (10% *v*/*v*) into MRS broth (pH 2.0, supplemented with 0.3% oxgall (*w*/*v*)) at 37 °C for 2 h. After incubation, the growth was monitored using the plate count method. The survival percentage was computed as: survival rate % = (numbers of living cells after incubation/numbers of initial living cells) × 100 [18].

#### 2.3.2. Tolerance to Bile

Tolerances to bile salt were performed as the method of Trivedi et al. [41]. Each strain overnight grown culture was inoculated (10% *v*/*v*) into MRS broth (containing 0.3% oxgall (*w*/*v*)) and incubated at 37 °C for 6 h. The survival percentage was calculated in Section 2.3.1.

#### 2.3.3. Tolerance to the Simulated Gastrointestinal (GI) Juice

Tolerances to the simulated gastrointestinal juice were examined in the light of procedures by Singh et al. [42]. Each strain was incubated in 10 mL sterile MRS broth for 24 h at 37 °C and mixed with 10 mL of sterile MRS broth with pH 2.0. The initial counts of samples were determined by spread plating on MRS agar plates and expressed as mean log colony-forming units per mL (CFU/mL). Then, samples were withdrawn after incubation for 1 h at 37 °C. Subsequently, four mL of ox-bile solution (10%) was added to each sample, followed by 17 mL of synthetic duodenum juice (6.4 g/L NaHCO_3_, 0.239 g/L KCl, 1.28 g/L NaCl, and pH-7.4). Each mixture was incubated at 37 °C, and the sample was withdrawn for viable counts after 2 h and 3 h, respectively. Viable counts were determined as above. The survival rate was calculated as (final (log_10_ CFU/mL))/(initial (log_10_ CFU/mL)) × 100.

Based on acid, bile, and simulated GI tolerance results above, strains with survivals over 50% were selected for subsequent experiments.

#### 2.3.4. Hydrophobicity Determination

Hydrophobicity was tested following the modified process of Saini and Tomar [43] using xylene and hexadecane, respectively. Each strain was cultured overnight and was centrifugated (12,000× *g* for 5 min, 5 °C), then cell sediment was harvested and washed thrice and resuspended in phosphate urea magnesium (PUM) buffer (0.2 g/L MgSO_4_·7H_2_O, 7.26 g/L KH_2_PO_4_, and 1.8 g/L Urea, pH 6.5), and the suspension reached absorbance (A_610nm_) of 0.8–1.0. Three milliliters of the suspension (pre-incubated at 37 °C for 10 min) was mixed with 1 mL of the hydrocarbons, vortexed for 120 s, and kept undisturbed at 37 °C for 1 h to allow complete phase separation. Then, the aqueous phase was removed carefully, and the absorbance (A_610nm_) was measured. The hydrophobicity percentage was computed using [(OD_initial_ − OD_final_)/OD_initial_] × 100.

#### 2.3.5. Aggregation Determination

The auto-aggregation capability was studied by the method of Collado et al. [44]. In short, an overnight grown culture of each strain was prepared, and the numbers of cell suspensions were adjusted to 10^7^–10^8^ CFU/mL. Then, the suspension was incubated at 37 °C for 1 d, and the absorbance (A_600nm_) was monitored. The auto-aggregation percentage was computed using [(1 − A_upper suspension_)/A_total strain suspension_] × 100.

The co-aggregation characteristic of each strain was evaluated as the procedure of Zuo et al. [45]. The preparation of cell suspension of each strain was the same as that of auto-aggregation determination. The cell suspension was mixed with an equal volume of every pathogen strain (described in Section 2.2.2), respectively, and the absorbance (A_600nm_) was measured (designated A_initial_). The mixture was incubated at 37 °C for 2 h, and the absorbance (A_600nm_) was measured again (designated A_final_). The percentage co-aggregation was calculated using [(A_initial_ − A_final_)/A_initial_] × 100.

### 2.4. Determination of Other Probiotic Properties

#### 2.4.1. Cholesterol Reduction

Cholesterol reduction activity was tested using the ophthalaldehyde method described by Shehata et al. [46]. Each strain was inoculated at 1% level into MRS broth (supplemented with 0.3% oxgall and water-soluble cholesterol (100 μg/mL)) and incubated at 37 °C for 24 h. Then, the culture was centrifugated (9000× *g*, 15 min), and the supernate was cell-free broth containing surplus cholesterol. One milliliter of KOH (33% *w*/*v*) and 2 mL of absolute ethanol were mixed with 1 mL of the cell-free broth, vortexed (60 s), heated (37 °C, 15 min), cooled thoroughly, followed by adding 2 mL of distilled water and 3 mL of hexane and vortexed again (60 s). One milliliter of the hexane layer was evaporated at 65 °C, and the surplus was immediately dissolved in 2 mL of o-phthalaldehyde and drastically mixed. Then, 0.5 mL concentrated sulphuric acid was added, followed by violently vortexed for 1 min. After 10 min, an absorbance value (A_550nm_) was read.

#### 2.4.2. Antibacterial Activity of Cell-Free Supernatants (CFS)

Each strain was cultured for 1 d in MRS broth, then centrifugated (4 °C, 10,000× *g*, 10 min), and the supernatant was filter sterilized (0.22 μm pore size) and used as CFS [47]. Five indicator strains (described in Section 2.2.3) were densely streaked on the Tryptone Glucose Extract (TGE) agar plates, respectively. After the agar plates were dry, different wells were made, and 50 μL of CFS was spotted. The plates were put at 37 °C for 1 d, and the inhabitation zone diameters were determined by a vernier caliper after incubation.

#### 2.4.3. Antioxidant Activity of Cell-Free Supernatants (CFS)

Each strain was inoculated into MRS broth. After 18 h, the culture was centrifugated (4 °C, 12,000× *g*, 10 min), and the cell precipitate was washed thrice and resuspended in phosphate buffer (cells with 10^8^–10^10^ CFU/mL), followed by ultrasonic disruption in ice bath for 15 min (3–8 s pulses). After ultrasonic disruption, the product was centrifugated (4 °C, 12,000× *g*, 10 min), and the supernate was CFS [48].

##### Tolerance to Hydrogen Peroxide

The tolerances to hydrogen peroxide (H_2_O_2_) were determined by the mini modified procedure of Zhang et al. [48]. Each strain was cultured overnight at 37 °C in MRS broth, and the precipitate was received by centrifugation (3000× *g*, 4 °C, 10 min). The precipitate was suspended at the level of 10^10^–10^8^ CFU/mL in PBS and incubated with 1 mM hydrogen peroxide at 37 °C for 8 h. At intervals of 2 h, the removed aliquots were spread onto MRS agar plates and cultured at 37 °C for 48 h. The number of each strain was expressed as mean log colony-forming units per ml (CFU/mL).

##### DPPH Radical Scavenging Activity

The DPPH (2,2-Diphenyl-1-picrylhydrazyl) radical scavenging activity of CFS was measured as the modified method [48]. A total of 0.8 mL of CFS of each strain was added into 1 mL of DPPH MeOH solution (0.2 mM) and thoroughly mixed. After 30 min, the absorbance (A_517nm_) was measured. The DPPH scavenging radical rate was calculated using [1 − (A_sample_/A_blank_)] × 100.

##### Hydroxyl Radical Scavenging Activity

The scavenging activity of hydroxyl radical of CFS from each strain was measured according to Zhang et al. [48]. A CFS sample was mixed with an equal volume of 1,10-phenanthroline (2.5 mM) and FeSO_4_ (2.5 mM). The reaction was initiated by adding an equal volume of H_2_O_2_ (20 mM) and incubating at 37 °C for 90 min. The absorbance value (A_536nm_) was monitored. Deionized water was a control. The scavenging property was calculated as: hydroxyl radical clearance rate % = [(A_sample_ − A_control_)/(A_d_ − A_control_)] × 100%. Where A_d_ refers to the absorbance of the solution without samples and H_2_O_2_.

### 2.5. Safety Assessment

#### 2.5.1. Antibiotic Sensitivity

The antibiotic sensitivity of each strain was tested by the disc diffusion method of the Clinical and Laboratory Standards Institute [49]. Seven kinds of antibiotics were divided into two groups by their mechanisms, i.e., protein synthesis suppressants (erythromycin, chloramphenicol, streptomycin, and tetracycline) and cytoderm suppressants (penicillin, ampicillin, and cefotaxime) [50]. The overnight grown culture of each strain was evenly coated on the surface of the MRS agar plate. The antibiotic discs containing penicillin (10 units), ampicillin (10 μg), cefotaxime (30 μg), erythromycin (15 μg), chloramphenicol (30 μg), and tetracycline (30 μg) were dispensed onto MRS agar aseptically. After incubation at 37 °C for 1 d to 2 d, the diameter (mm) of inhibition zones of each plate was measured by a vernier caliper, and the results were expressed as resistant (≤15 mm), intermediate (15–21 mm), or susceptible (≥21 mm) [51].

#### 2.5.2. Hemolytic Activity

Hemolytic activity was researched by the procedure of Park et al. [52]. Overnight grown culture of each strain was streaked onto sterile blood agar plates (adding 5% sheep blood) and incubated at 37 °C for 1 d. Strain exhibiting a green or a clear halo surrounding the colony was designated as α-hemolysis or β-hemolytic, respectively, which was harmful to the host. Strains forming no zone (designated as no-hemolysis or γ-hemolytic) were safe and selected as probiotics [53]. *Staphylococcus aureus* was a positive control.

#### 2.5.3. Gelatinase Hydrolysis

The Nutrient Gelatine stab method was used to perform the gelatinase activity. The inoculum of the 24–48 h old test strain was stab-inoculated into tubes containing Nutrient Gelatine medium, then incubated at 37 °C for 7 d and checked every day. The media were immersed in an ice bath for 15–30 min to observe liquefaction [52]. *Escherichia coli* ATCC25922 were positive control.

#### 2.5.4. Biogenic Amine (BA) Production

The selected strains detected the abilities producing biogenic amine using the method of Joosten and Northolt [54]. A total of 0.5 mL of the selected cultures were transferred twice in succession into MRS broth supplemented with pyridoxal-5-phosphate at 0.005% (*w*/*v*) and each one of the biogenic amine precursors at 0.1% (*w*/*v*): tyrosine monohydrochloride (for tyramine), histidine monohydrochloride (for histamine), lysine monohydrochloride (for lysine), and ornithine monohydrochloride (for putrescine) (Macklin). All cultures were incubated at 37 °C for 24–48 h, followed by streaking onto decarboxylase agar supplemented with each biogenic amine precursor 1% (*w*/*v*) mentioned above. The plates were incubated at 37 °C for 3–7 days, and color changes from yellow to purple were positive results [55]. *Escherichia coli* ATCC25922 was a positive control.

Based on these results, strains without hemolytic activity, gelatinase hydrolysis, and BA formation activity were selected for identification.

### 2.6. Identification of Selected Probiotics by 16S rRNA Gene Sequencing

The universal primer pair 27F-1492R [56] was used to amplify the 16S rRNA gene of selected strains in 2.5. The PCR products were sequenced by Sangon (Beijing, China). Sequence results of strains were aligned using the BLAST algorithm in the NCBI database. The sequences were submitted to the GenBank of NCBI to receive the accession numbers.

### 2.7. Experimentation and Analysis

All experiments were repeated independently three times, the data were represented as mean ± standard deviation, and were analyzed using SPSS (version 20.0). The significant differences among means were determined by Duncan’s multiple range test [57] at the 5% level.

## 3. Results and Discussions

### 3.1. Morphological and Physicochemical Characteristics of LAB

In total, 18 LAB strains were obtained from sour porridge upon the distinct morphological features on MRS agar plates, and their physicochemical features were listed in Table 1. All of them were round, white or milk white, rod-shape, or short rod-shape, Gram-positive, non-sporulating, catalase-negative, oxidase-negative, nitrate reductase-negative. Eighteen LAB strains had the potential to ferment various saccharides. 

### 3.2. Survival Rates of LAB in Simulated Gastrointestinal Conditions

#### 3.2.1. Tolerance to Acid

Before probiotics play benefits to the host, they must stand the low pH in the GI condition [58]. The survival rates of 18 strains under the acidic condition are shown in Table 2. Low pH drastically affected the survival rates of all strains, which was in agreement with most reports about LAB [56,59,60]. All strains could variously tolerate the acidic condition, and most of them (14 out of 18) showed above 50% survival rates. The findings were higher than others [40,59] but were approximately equal to the findings by Aarti et al. [56]. Meanwhile, it was very surprising that the survival rates of six strains (L2, L3, L5, L16, L17, and L18) increased obviously during the acid condition, which was similar to results by Oh and Jung [60]. Acid resistance of *Lactobacillus* was due to converting amino acids into biogenic amines [61,62]. Abushelaibi et al. [63] found that the tolerance to acid was strain- or species-dependent, whereas Arena et al. [64] proved that it seemed to be strain-dependent rather than species-specific.

#### 3.2.2. Tolerance to Bile

The bile content in human was between 0.3% and 0.5% [65]. Therefore, it was very necessary to assess the effect of 0.3% bile on the growth of LAB. In the small intestine, the retention time of food is about 4–6 h [18]. In this study, 18 strains exhibited different abilities of bile tolerance against 0.3% oxgall for 6 h, and half of them showed above 50% survival rates (Table 2). Aarti et al. [56] demonstrated that *Levilactobacillus brevis* could resist bile during 0–48 h, and the maximum number appeared at 36 h. Oh and Jung [60] had discovered that *Lactobacillus* had 89–95% survival rates at 0.3% oxgall for 6 h. In the study by Abushelaibi et al. [63], 91% of strains exhibited 50% survival rates. Saini and Tomar [43] reported that survival rates of two *Lactobacillus plantarum* cultures were above 75% after 3 h at 1% bile. In this study, it was amazing that strain L14 exhibited the maximum tolerance (115%), which was similar to that by Oh and Jung [60], and this result was very unusual and different from most other findings. The bile tolerance of bacteria was speculated to be the existence of several proteins [66]. In addition, compared to acid stress, bile stress has stronger inhibition of *Lactobacillus* [43].

#### 3.2.3. Tolerance to Simulated Gastrointestinal (GI) Juice

As the two stresses of stomach transit and small intestinal transit might interact and thereby affect the viability of the strains in a synergistic fashion, it is important to evaluate all components (low pH, bile salts, and duodenum juice) in one system rather than evaluating the effect of each component in separate experiments [42]. So, probiotics must survive after passing through the GI tract [65]. Among 18 LAB strains, 44% of strains exceeded 50% survival rates exposed to simulated GI juice for 3 h, and strain L10 exhibited the maximum tolerance (78%), followed by strains L18 and L17 (Table 2). These results were far higher than most *Lactobacillus reuteri* isolated from human breast-fed infants, in which the maximal survival rate was 70.81%, and the survival rate of *Lactobacillus* *reuteri* ATCC 55730 was 64.14% [42]. These results in this paper suggest that isolates from the sour porridge could successfully pass the human stomach and reach the intestinal and function effectively. Simulated GI juice tolerance of *Lactobacillus* may be attributed to biogenic amines produced by the strain itself [67].

Based on acid, bile, and simulated GI juice tolerance results above, eight strains (L3, L10, L12, L13, L14, L16, L17, and L18) were selected for subsequent experiments because their survival rates were over 50%.

#### 3.2.4. Hydrophobicity Assay

The capacity of probiotics to attach to hydrocarbons is a measure of their capacity to adhere to the epithelium of the digestive tract [68], which finally determines its capacity for colonization in the gut. The selected 8 strains showed different levels of hydrophobicities against xylene and hexadecane (Table 3). In the case of xylene, strains L10, L13, and L18 showed higher hydrophobicity percentages, varying from 30% to 44%. Similarly, for hexadecane, higher affinities appeared in strains L10, L13, L16, and L18, ranging from 30% to 39%. These results were higher than those of LAB [39,69]. Abushelaibi et al. [61] found that two out of nine strains had higher hydrophobicity against xylene but lower hydrophobicity toward hexadecane. In this paper, strain L10 consistently possessed maximum hydrophobicity toward xylene and hexadecane, which exceeded the highest hydrophobicity of *Lactobacillus casei* [70]. Hydrophobicity of probiotics differed not only between sibling species but also among the same species [71]. The reason for differences in hydrophobicity among different strains may be the diversification in hydrophobic/hydrophilic contents in the cytoderm.

#### 3.2.5. Aggregation Assay

Auto-aggregation could reflect the capacity of the probiotics’ adhesion to epithelial cells of the GI tract in humans [72]. Therefore, the higher auto-aggregation, the stronger adhesion. The auto-aggregation percentage of selected 8 strains ranged from 15% to 39% (Table 3), which were higher than other strains researched by Ayyash et al. [39]. Among the selected 8 strains, the most auto-aggregative strain was L10 (39%), which was comparable to the result of *Lactobacillus reuter* (38.8%) reported by Abushelaibi et al. [63]. Angmo et al. [59] found that the auto-aggregation percentages of *Levilactobacillus brevis* ranged from 5.04% to 64.78%. Much research demonstrated that co-aggregation could promote colonization of the GI tract [73]. The co-aggregation percentages of selected 8 strains with G^+^ pathogens were higher than those for G^−^ pathogens (Table 4), which agreed with the results of Ayyash et al. [39], but were opposite to the report by Abushelaibi et al. [63]. Overall, strain L10 and L14 demonstrated higher co-aggregation abilities than others. The co-aggregation ratios of probiotics were strain-specific dependent [44]. The co-aggregation characteristics may be attributed to the cell surface constituents, such as interactions between proteinaceous components and carbohydrate-lectin [74], or the LAB form a defensive barrier preventing the colonization of pathogens in the GI tract [75].

### 3.3. Other Probiotics Properties

#### 3.3.1. Cholesterol Reduction

**Figure 1:**
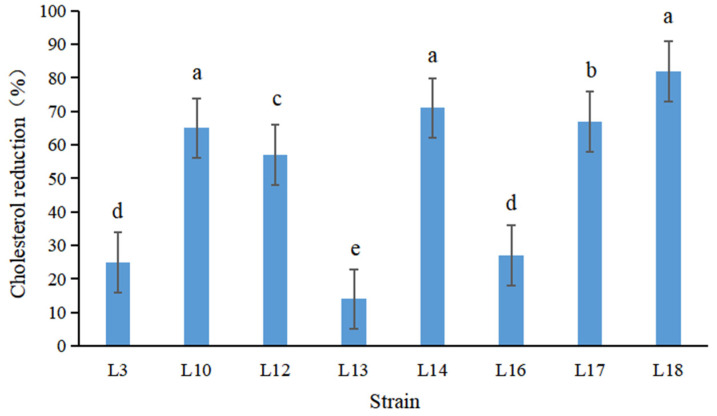
The cholesterol reduction (%) of selected 8 strains. The different letters indicated significant difference among treatments (*p* < 0.05).

The high content of cholesterol in the blood is a main factor of risk, especially for coronary artery patients. Therefore, it is very vital and necessary to decrease cholesterol content in serum. Cholesterol reduction activity is an indispensable feature in the selection of potential probiotics. The selected 8 strains showed varying degrees of cholesterol reduction levels ranging between 14% and 81% (Figure 1). Remarkably, strains L18, L14, L17, L10, and L12 were significantly more efficient. Strain L10 and L18 manifested maximum abilities (80% and 81%), which was higher than *Lactobacillus acidophilus* studied by Abushelaibi et al. [63] and Ayyash et al. [39]. The mechanisms of cholesterol reduction were proposed as decomposition of the enzyme, adhesion to the cytoderm, and incorporation in the cytoderm [76].

#### 3.3.2. Antibacterial Activity of Cell-Free Supernatants (CFS)

The selected 8 strains showed varying inhibition zones against G^+^ and G^−^ bacteria, which almost agreed with those of Angmo et al. [59], and strain L10 exhibited the maximum inhibition zone (Table 4). Interestingly, all strains had greater influences against *S. aureus* than other pathogens, which was in line with findings by Abushelaibi et al. [63]. Compared with the research by Adesulu-Dahunsi et al. [17], the antibacterial activities against *B. cereus* were lower, whereas those against *E. coli* and *S. aureus* were higher. The antibacterial substances of CFS were presumed to be organic acids, carbon dioxide, bacteriocin-like compounds, antimicrobial peptides, and H_2_O_2_ [77,78].

#### 3.3.3. Antioxidant Activity of Cell-Free Supernatant (CFS)

##### Tolerance to Hydrogen Peroxide

**Figure 2:**
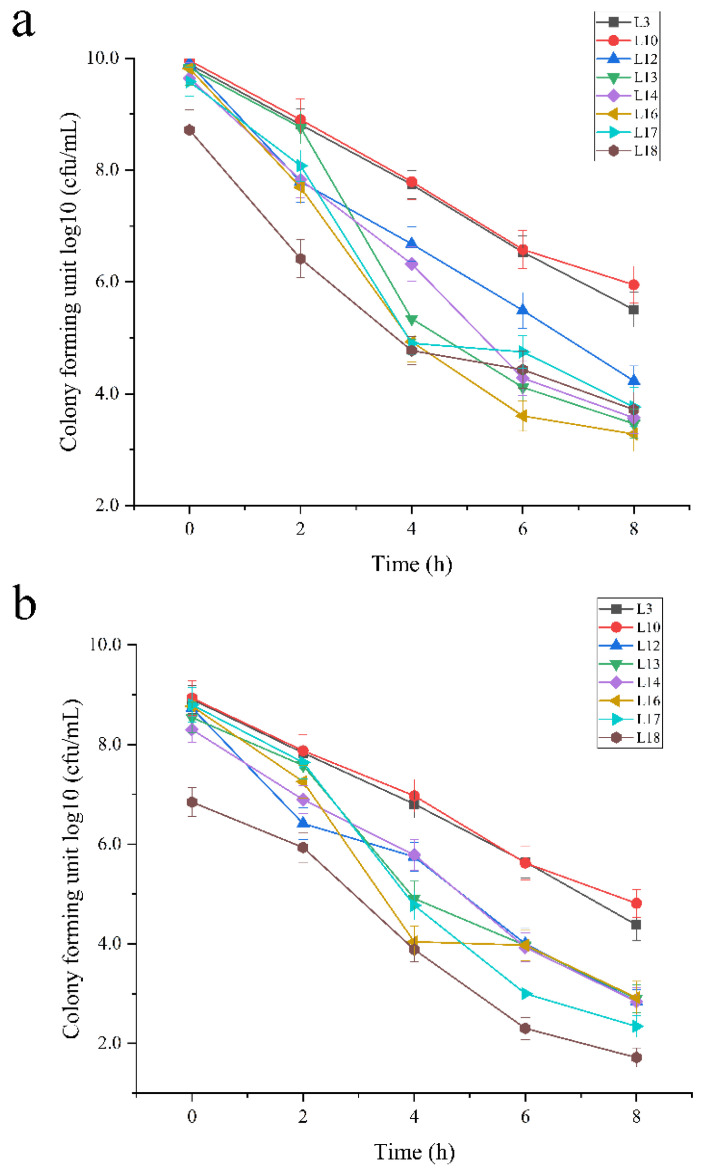

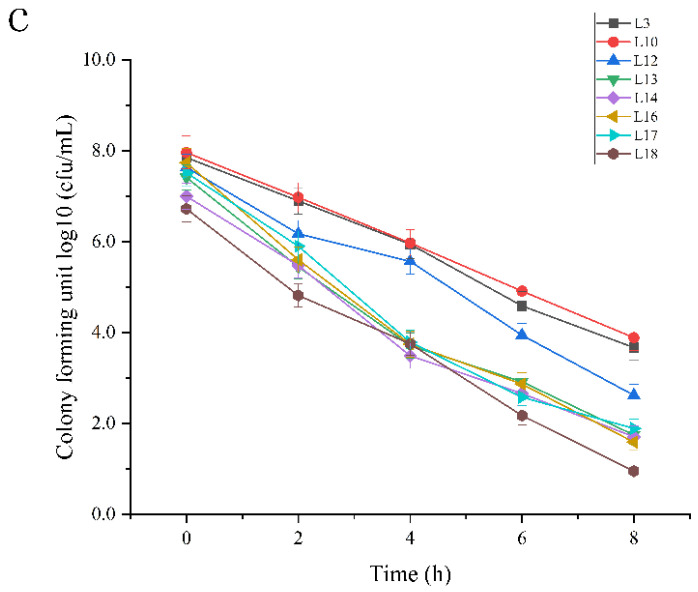
Viable numbers [Log_10_ CFU/mL] of selected 8 strains after being cultured with H_2_O_2_ for different times. The initial cell concentration of 8 strains was 10^10^ CFU/mL in (**a**), 10^9^ CFU/mL in (**b**) and 10^8^ CFU/mL in (**c**), respectively.

The tolerance to H_2_O_2_ of each strain became weaker with the decrease in initial bacterial cell concentration, i.e., the tolerances of initial cells with 10^10^ CFU/mL were the strongest (Figure 2a), whereas tolerances of initial cells with 10^8^ CFU/mL were the weakest (Figure 2c). These findings were matched with studies by Aarti et al. [56]. The selected 8 strains all showed significantly variant tolerances to H_2_O_2_ for each concentration of cells (*p* < 0.05), and the number of viable cells of strains L3 and L10 after 8 h was the highest among them. This indicates that strains L3 and L10 had the best tolerances to H_2_O_2_. These results were higher than those of Zhang et al. [48].

##### DPPH Radical Scavenging Activity

**Figure 3:**
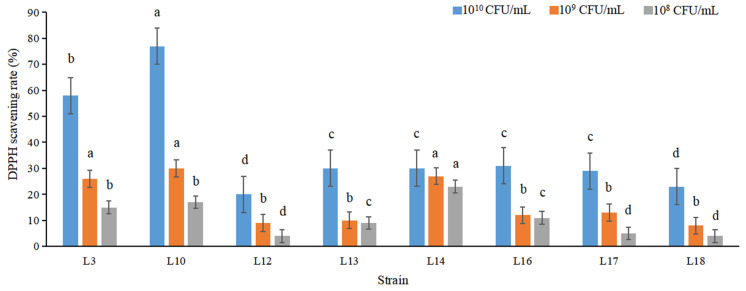
The DPPH radicals scavenging rate (%) of selected 8 strains. The different letters indicated significant difference among treatments (*p* < 0.05).

It is generally recognized that probiotics with antioxidant potential are good for health [79,80]. The DPPH radical scavenging activity of cells with 10^10^ CFU/mL was higher than those of 10^8^ and 10^9^ CFU/mL of selected 8 strains (Figure 3). Therefore, the DPPH radical scavenging potential increased in a concentration-dependent manner, and the same results were achieved by Aarti et al. [56]. Cells with 10^10^ CFU/mL of all strains had different DPPH scavenging activities ranging from 77% to 20%, and strain L10 exhibited peak activity (77%). Our results were higher than those of Aarti et al. [56].

##### Hydroxyl Radical Scavenging Activity

**Figure 4:**
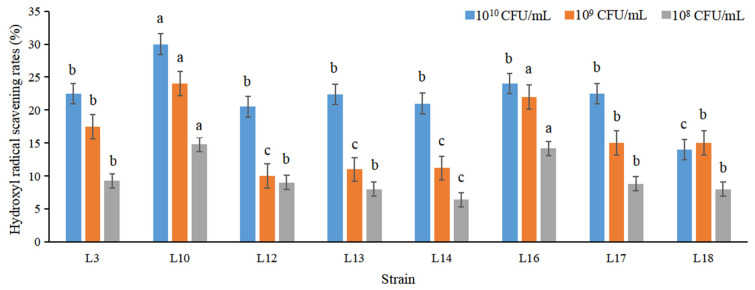
The hydroxyl radical scavenging rate (%) of selected 8 strains. The different letters indicated significant difference among treatments (*p* < 0.05).

It is the same as the DPPH scavenging activities. The hydroxyl radical scavenging potentials of selected 8 strains also increased in a concentration-dependent mode (Figure 4). The hydroxyl radical clearance activities of all strains (cells with 10^10^ CFU/mL) ranged from 20.46% to 27.58%, which was close to those by Sharma et al. [81]. The disparities in scavenging potentials of different strains may be attributed to different mechanisms [82], and exopolysaccharide was the antioxidant components [83].

### 3.4. Safety Assessment

#### 3.4.1. Antibiotic Sensitive

Probiotics showing sensitivity toward antibiotics are preferable [39]. Antibiotic-sensitive probiotics could not horizontally transmit antibiotic-resistant genes to pathogens [84]. Much research has revealed that the sensitivities of LAB to different antibiotics depend on the species, and these susceptibilities vary several-fold between species [2,85]. However, the antibiotic resistance of L13, L14, L16, and L17 was highly consistent in this paper (Table 5). All of the selected 8 strains were sensitive to ampicillin and penicillin, which was in accord with the results by Xia et al. [51] but was a little different from those of Abushelaibi et al. [63]. The selected 8 strains were all sensitive to cefotaxime, analogously, all LAB were sensitive to cefamezin [51], and cefotaxime and cefamezin had the same structure with the same bactericidal mechanism. Most isolates (except L12) were alive to erythromycin in this study, Hashemi et al. [86] and Abushelaibi et al. [63] also found that most of LAB were moderate susceptibility to erythromycin. In this paper, 62.5% of isolates were susceptible to chloramphenicol, and 75% of isolates were susceptible to tetracycline, while all lactobacilli strains showed susceptibility or moderate susceptibility [86].

#### 3.4.2. Hemolytic, Gelatinase Hydrolysis Activity, and Biogenic Amine Production

The introduction of a new probiotic strain demands that it has no hemolytic and biogenic amine production activity. Among the selected 8 strains, strains L3, L12, and L13 showed β-hemolysis, and L12 and L13 could produce biogenic amine. In the research of Park et al. [52], all *Levilactobacillus* *brevis* strains produced tyramine and/or histamine from tyrosine or histidine, which was very different from this study. None of them showed gelatinase hydrolysis activity. Therefore, five strains (L10, L14, L16, L17, and L18) were safe and thus selected as probiotics [59].

### 3.5. Identification of Selected Probiotics by 16S rRNA Gene Sequencing

The five strains (L10, L14, L16, L17, and L18) selected in Section 3.3.2 were identified by 16S rRNA gene sequencing. The blast results showed that they were all similar to *Levilactobacillus brevis* (Table 6). This indicated that more molecular techniques were needed to achieve better results in the next work. The accession numbers of L10, L14, L16, L17, and L18 were MN267485, MN267486, MN267487, MN267488, and MN267489, respectively.

## 4. Conclusions

This is the first study focused on the probiotic characteristics of LAB isolated from naturally fermented sour porridge with broomcorn millet in northwestern Shanxi, China. In this study, five strains (*Levilactobacillus brevis*) were selected as potential probiotics because of their higher survival rates in the simulated GI tract, better cholesterol reduction percentages, stronger antibacterial, and antioxidative capabilities, safeties trust such as antibiotic-sensitive, non-hemolysis, non-gelatinase hydrolysis and without biogenic amine producing. Among them, strain L10 was particularly noteworthy. In further research, strain L10 could be explored in the following aspects: (i) used as a starter in the fermentation of sour porridge or other fermented foods to enhance the taste, look and crunchiness, extend the shelf life, etc.; (ii) used to develop functional foods with lower cholesterol effect, antibacterial effect, antioxidative effect, and so on; (iii) applied as probiotic to maintain body health such as modulate the balance of gut microbiota, decrease blood glucose, reduce blood pressure, keep the mouth healthy, etc.; (iv) used in the beauty products; (v) used in the animal feeds to improve the production traits and so forth.

## Figures and Tables

**Table 1:** Morphological and physicochemical results of 18 LAB strains.

Tests	Strains																	
	L1	L2	L3	L4	L5	L6	L7	L8	L9	L10	L11	L12	L13	L14	L15	L16	L17	L18
Colony shape	Round	Round	Round	Round	Round	Round	Round	Round	Round	Round	Round	Round	Round	Round	Round	Round	Round	Round
Colony color	White	Milk white	White	White	Light white	White	Milk white	White	White	White	white	White	White	Light white	White	White	White	Milk white
Shape	Rod	Rod	Rod	Rod	Rod	Rod	Rod	Rod	Rod	Short Rod	Rod	Rod	Short Rod	Short Rod	Rod	Short Rod	Short Rod	Rod
Gram staining	+	+	+	+	+	+	+	+	+	+	+	+	+	+	+	+	+	+
Endospore	Non- spore	Non- spore	Non- spore	Non- spore	Non- spore	Non- spore	Non- spore	Non- spore	Non- spore	Non- spore	Non- spore	Non- spore	Non- spore	Non- spore	Non- spore	Non- spore	Non- spore	Non- spore
Catalase	-	-	-	-	-	-	-	-	-	-	-	-	-	-	-	-	-	-
Oxidase	-	-	-	-	-	-	-	-	-	-	-	-	-	-	-	-	-	-
Nitrate reductase	-	-	-	-	-	-	-	-	-	-	-	-	-	-	-	-	-	-
Amygdalin	+	-	+	+	-	-	+	+	+	-	+	-	-	-	+	-	-	-
Arabinose	+	+	-	+	+	-	-	+	-	+	+	+	-	+	-	+	+	+
Cellose	+	+	+	+	-	+	+	+	+	-	+	+	+	-	+	-	-	-
Esculin	+	-	+	+	-	-	+	+	+	+	+	+	+	-	+	+	-	+
Fructose	+	+	+	+	+	+	+	+	+	+	+	+	+	+	+	+	+	+
Galactose	+	+	+	+	+	+	+	+	+	+	+	+	+	+	+	-	-	-
Glucose	+	+	+	+	+	+	+	+	+	+	+	+	+	+	+	+	+	+
Gluconate	+	+	+	+	+	+	-	+	+	+	+	+	+	+	+	+	+	+
Lactose	+	+	+	+	+	+	+	+	+	+	+	+	+	+	+	-	+	-
Maltose	+	+	+	+	+	+	+	+	+	+	+	+	+	+	+	+	+	+
Mannitol	+	-	+	+	-	-	-	+	+	-	+	+	+	-	+	-	-	-
Mannose	+	+	+	+	+	+	+	+	+	-	+	+	+	-	+	-	-	-
Melezitose	+	-	+	-	-	-	-	+	+	-	-	-	-	-	-	-	-	-
Melibiose	+	+	+	+	+	+	+	+	+	+	+	+	+	+	+	+	+	+
Raffinose	-	+	-	-	+	+	+	-	-	+	-	-	-	-	-	+	+	+
Rhamnose	+	-	+	+	-	-	-	+	+	-	+	+	+	-	+	-	-	-
Ribose	+	+	+	+	+	+	-	+	+	+	+	+	+	+	+	+	+	+
Salicin	+	-	+	+	-	-	+	+	+	-	+	+	+	-	+	-	-	-
Sorbitol	+	-	+	+	-	-	-	+	+	-	+	+	+	-	+	-	-	-
Sucrose	+	+	+	+	+	+	+	+	+	+	+	+	+	+	+	-	+	+
Mycose	+	+	+	+	+	+	+	+	+	-	+	+	+	-	+	-	-	-
Xylose	+	+	+	+	+	+	-	-	+	+	-	-	-	+	+	+	+	-

‘+’ positive; ‘-’ negative.

**Table 2:** Acid, bile, and gastrointestinal juice tolerance of 18 strains.

Strains	Survival Rate (%)			
	Acid	Bile	Gastrointestinal Juice	
			2 h	3 h
L1	27 ± 0.67 ^n^	38 ± 0.06 ^a^	28 ± 0.41 ^fg^	17 ± 0.23 ^h^
L2	215 ± 0.17 ^b^	44 ± 0.07 ^a^	10 ± 0.02 ^i^	7 ± 0.06 ^j^
L3	171 ± 0.49 ^c^	68 ± 0.03 ^cd^	60 ± 0.19 ^c^	54 ± 0.14 ^e^
L4	16 ± 0.20 ^p^	37 ± 0.01 ^a^	28 ± 0.25 ^fg^	11 ± 0.31 ^ij^
L5	99 ± 0.57 ^f^	36 ± 0.08 ^a^	32 ± 0.14 ^ef^	19 ± 0.18 ^gh^
L6	81 ± 0.24 ^h^	45 ± 0.01 ^a^	37 ± 0.60 ^de^	28 ± 0.45 ^f^
L7	72 ± 0.31 ^i^	39 ± 0.04 ^a^	40 ± 0.07 ^d^	29 ± 0.46 ^f^
L8	14 ± 0.36 ^q^	40 ± 0.03 ^a^	32 ± 0.33 ^ef^	16 ± 0.27 ^hi^
L9	61 ± 0.09 ^k^	41 ± 0.05 ^a^	21 ± 0.05 ^h^	9 ± 0.58 ^j^
L10	86 ± 0.66 ^g^	80 ± 0.06 ^d^	86 ± 0.52 ^a^	78 ± 0.11 ^a^
L11	50 ± 0.57 ^m^	44 ± 0.07 ^a^	30 ± 0.04 ^fg^	24 ± 0.04 ^fg^
L12	59 ± 0.33 ^l^	59 ± 0.16 ^bc^	64 ± 0.31 ^c^	50 ± 0.16 ^e^
L13	87 ± 0.25 ^g^	56 ± 0.04 ^bc^	62 ± 0.62 ^c^	50 ± 0.87 ^e^
L14	64 ± 0.18 ^j^	115 ± 0.19 ^e^	75 ± 0.09 ^b^	55 ± 0.11 ^de^
L15	22 ± 0.32 ^o^	60 ± 0.22 ^bc^	24 ± 0.39 ^gh^	8 ± 0.77 ^j^
L16	120 ± 0.32 ^e^	64 ± 0.10 ^c^	76 ± 0.15 ^b^	60 ± 0.80 ^cd^
L17	131 ± 0.45 ^d^	74 ± 0.08 ^cd^	84 ± 0.78 ^a^	65 ± 1.11 ^bc^
L18	245 ± 0.28 ^a^	75 ± 0.03 ^cd^	83 ± 0.29 ^a^	70 ± 0.56 ^b^

Data in the same column with different subscript letters indicate significant differences at *p* < 0.05.

**Table 3:** Hydrophobicity and aggregation of the selected 8 strains.

Strains	Hydrophobicity (%)			Aggregation (%)			
	Xylene	Hexadecane	Auto- Aggregation (%)	Co-Aggregation (%)			
				*L. monocytogenes*	*S. aureus*	*E. coli*	*S. typhimurium*
L3	20 ± 0.23 ^c^	23 ± 0.33 ^d^	21 ± 0.54 ^e^	6.65 ± 0.08 ^e^	7.08 ± 0.04 ^e^	6.18 ± 0.05 ^d^	6.22 ± 0.05 ^e^
L10	42 ± 0.07 ^a^	39 ± 0.10 ^a^	39 ± 0.56 ^a^	18.13 ± 0.09 ^a^	10.40 ± 0.21 ^a^	16.23 ± 0.33 ^a^	9.77 ± 0.02 ^a^
L12	24 ± 0.45 ^c^	20 ± 1.08 ^d^	19 ± 0.38 ^f^	8.74 ± 0.03 ^c^	9.76 ± 0.02 ^b^	8.11 ± 0.02 ^c^	9.75 ± 0.03 ^a^
L13	32 ± 0.62 ^b^	30 ± 0.05 ^c^	26 ± 0.51 ^c^	3.41 ± 0.04 ^g^	3.28 ± 0.04 ^g^	3.39 ± 0.12 ^f^	2.39 ± 0.02 ^g^
L14	21 ± 0.16 ^c^	14 ± 0.44 ^e^	15 ± 0.29 ^g^	11.04 ± 0.03 ^b^	9.94 ± 0.02 ^b^	10.47 ± 0.26 ^b^	9.57 ± 0.05 ^b^
L16	20 ± 0.15 ^c^	31 ± 0.97 ^bc^	23 ± 0.31 ^d^	4.69 ± 0.15 ^f^	5.25 ± 0.04 ^f^	4.60 ± 0.17 ^e^	4.38 ± 0.01 ^f^
L17	21 ± 0.38 ^c^	19 ± 0.08 ^d^	21 ± 0.34 ^ef^	7.94 ± 0.03 ^d^	7.44 ± 0.03 ^d^	7.85 ± 0.03 ^c^	7.12 ± 0.02 ^d^
L18	30 ± 0.74 ^b^	35 ± 1.31 ^ab^	33 ± 0.25 ^b^	8.66 ± 0.03 ^c^	8.12 ± 0.06 ^c^	8.28 ± 0.01 ^c^	7.92 ± 0.03 ^c^

Data in the same column with different subscript letters indicate significant differences at *p* < 0.05.

**Table 4:** Antibacterial activity of cell-free supernatants (CFS) of selected 8 strains.

Strains	Inhibition Zone Diameter (mm)				
	G^+^ Bacteria				G^−^ Bacteria
	*B. cereus*	*E. faecium*	*L. monocytogens*	*S. aureus*	*E. coli*
L3	5.34 ± 0.10 ^d^	5.21 ± 0.22 ^d^	10.02 ± 0.12 ^d^	17.97 ± 0.04 ^e^	14.72 ± 0.13 ^f^
L10	10.12 ± 0.11 ^a^	10.25 ± 0.13 ^a^	15.01 ± 0.23 ^a^	26.40 ± 0.21 ^a^	23.65 ± 0.10 ^a^
L12	3.87 ± 0.23 ^e^	3.85 ± 0.35 ^e^	10.30 ± 0.19 ^d^	17.75 ± 0.07 ^e^	18.57 ± 0.03 ^d^
L13	2.88 ± 0.18 ^f^	2.98 ± 0.26 ^f^	10.39 ± 0.27 ^d^	16.48 ± 0.04 ^f^	13.55 ± 0.06 ^g^
L14	8.24 ± 0.02 ^b^	8.03± 0.19 ^b^	13.03 ± 0.22 ^b^	20.52 ± 0.16 ^c^	20.19 ± 0.02 ^c^
L16	6.36 ± 0.16 ^c^	6.55 ± 0.50 ^c^	11.47 ± 0.20 ^c^	19.74 ± 0.04 ^d^	18.48 ± 0.02 ^d^
L17	1.43 ± 0.14 ^g^	2.91 ± 0.49 ^f^	13.83 ± 0.15 ^b^	15.26 ± 0.01 ^g^	15.56 ± 0.02 ^e^
L18	6.28 ± 0.47 ^c^	6.69 ± 0.27 ^c^	10.22 ± 0.05 ^d^	24.46 ± 0.01 ^b^	22.71 ± 0.01 ^b^

Data in the same column with different subscript letters indicate significant differences at *p* < 0.05.

**Table 5:** Antibiotic susceptibility of selected 8 strains.

Antibiotics	Strains							
	L3	L10	L12	L13	L14	L16	L17	L18
Penicillin	S ^a^	S	S	S	S	S	S	S
Ampicillin	S	S	S	S	S	S	S	S
Cefotaxime	S	S	S	S	S	S	S	S
Erythromycin	S	S	R	S	S	S	S	S
Chloramphenicol	R ^b^	S	R	S	S	S	S	R
Tetracycline	S	R	S	S	S	S	S	R

^a^ Susceptible; ^b^ resistant.

**Table 6:** Blast results of selected 5 strains by 16S rRNA gene sequence.

Strains	Accession No.	The Most Similar Sequence (Accession No.)	Similarity (%)
L10	MN267485	*Levilactobacillus brevis* (MT604645)	99.93
L14	MN267486	*Levilactobacillus brevis* (MT604645)	100
L16	MN267487	*Levilactobacillus brevis* (MT613460)	100
L17	MN267488	*Levilactobacillus brevis* (MN267488)	100
L18	MN267489	*Levilactobacillus brevis* (MT515953)	100
